# Molecular characterization of a selected cohort of patients affected by pulmonary metastases of malignant melanoma: Hints from BRAF, NRAS and EGFR evaluation

**DOI:** 10.18632/oncotarget.4503

**Published:** 2015-07-04

**Authors:** Alessandra Ulivieri, Giuseppe Cardillo, Liborio Manente, Gregorino Paone, Andrea Petricca Mancuso, Leonardo Vigna, Enrico Di Stasio, Rita Gasbarra, Salvatore Girlando, Alvaro Leone

**Affiliations:** ^1^ Anatomic Pathology Unit, San Camillo-Forlanini Hospitals, Rome, Italy; ^2^ Laboratory of Biomedical research “Fondazione Niccolò Cusano per la Ricerca Medico-Scientifica” Niccolò Cusano University of Rome, Rome, Italy; ^3^ Thoracic Surgery Unit, San Camillo-Forlanini Hospitals, Rome, Italy; ^4^ Department of Respiratory Diseases, San Camillo-Forlanini Hospitals, Rome, Italy; ^5^ Department of Medical Oncology, San Camillo-Forlanini Hospitals, Rome, Italy; ^6^ Institute of Biochemistry and Clinical Biochemistry, Università Cattolica del Sacro Cuore, Rome, Italy; ^7^ Anatomic Pathology Unit, S. Chiara Hospital, Trento, Italy

**Keywords:** Pathology, melanoma, pulmonary metastases, BRAF, NRAS, EGFR

## Abstract

**Background:**

Melanoma is highly curable in early stages but holds devastating consequences in advanced phases with a median survival of 6–10 months. Lungs are a common metastasis target, but despite this, limited data are available on the molecular status of pulmonary lesions.

**Materials and Methods:**

25 patients with surgically resected melanoma lung metastases were screened for BRAF, NRAS, CKIT and EGFR alterations. The results were correlated with time to lung metastasis (TLM), relapse-free survival after metastasectomy (RFS) and overall survival (OS).

**Results:**

BRAF or NRAS were mutated in 52% and 20% of cases while CKIT was unaffected. Chromosome 7 polysomy was detected in 47% of cases with 17.5% showing EGFR amplification and concomitant BRAF mutation. NRAS mutated patients developed LM within 5 yrs from primary melanoma with larger lesions compared with BRAF (mean diameter 3.3 ± 2.2cm *vs* 1.9 ± 1.1cm, *p* = 0.2). NRAS was also associated with a shorter median RFS and OS after metastasectomy. Moreover, Cox regression analysis revealed that NRAS mutation was the only predictive factor of shorter survival from primary melanoma (*p* = 0.039, OR = 5.5 (1.1–27.6)).

**Conclusions:**

Molecular characterization identifies advanced melanoma subgroups with distinct prognosis and therapeutic options. The presence of NRAS mutation was associated to a worse disease evolution.

## INTRODUCTION

Melanoma is a highly curable malignancy in early stages but unfortunately holds devastating consequences when metastases develop, with a decline in 5-year survival rate from 98% for localized to only 16% for metastatic melanoma [1]. The site of distant metastasis is an important independent predictor of survival with patients harboring visceral melanoma metastases showing the worst prognostic behavior [1, 2]. Lung is the second most common site for metastatic spread and the annual probability of developing lung metastatic melanoma (LMMs) progressively increases from 10% at 5 years to 17% at 15 years after the resection of the primary tumor [3]. Until recently, systemic chemotherapies (dacarbazine), hydroxyurea, or immunotherapy with high-dose interleukin-2 (IL-2) were the only treatment options approved by the U.S. Food and Drug Administration (FDA) for patients with advanced melanoma [4–6]. These systemic treatments provide little, if any, attested survival benefit for patients harboring LMM, with surgery remaining, unfortunately only for few eligible patients, the best treatment to improve overall survival (OS) [7, 8]. Recent progresses in the understanding of melanoma pathogenesis have allowed the identification of both immunotargeting agents, as ipilimumab, and actionable driver mutations in several genes potentially exploitable for therapeutic purposes [9–11]. The BRAF gene, encoding for a protein member of the mitogen-activated-protein-kinase family (MAPK) [12], is mutated in approximately half of all melanomas, and its mutations, commonly occurring at codon 600 (BRAF^V600^) [9, 13], have been exploited to develop drugs (vemurafenib, dabrafenib and trametinib) effective against BRAF-driven metastatic melanoma [13–17]. In addition to BRAF, NRAS mutations, mostly affecting codon 61, are found in 10–15% of melanomas and are involved in mutagenic activation of the MAPK pathway [18–20]. They can be targeted by MEK162, a strong MEK1/2-inhibitor [21]. Melanomas have also been reported to over express CKIT whose mutations are found in 1.7% of cutaneous melanomas, 23% of acral melanomas, and 15.6% of mucosal melanomas [22, 23]. Emerging evidence suggests that melanomas with CKIT activation may respond to specific targeted agents [24–26]. Finally, epidermal-growth-factor receptor (EGFR) copy number alterations have been found in primary cutaneous malignant melanomas and their presence has been associated with poor prognosis [27, 28].

Despite of the observation that the lungs are a frequent site of visceral metastases from melanoma, very limited and scattered data are available on the molecular status of pulmonary lesions. The molecular characterization of the above-mentioned genes in a lung metastatic setting could help in the decision to select the best possible therapeutic option among novel tailored biologic therapies or surgical treatment. Based on the above observations, the aim of the present study was to determine the frequency of alterations of the aforementioned genes and possible clinical and pathological correlations in a well characterized cohort of patients affected by lung metastases from advanced melanoma.

## RESULTS

### Patients and samples characteristics

A total of 25 lung metastasis specimens collected after surgery from 25 patients with LMM were analyzed. The cohort included 10 females (40%) and 15 males (60%). Median age at first diagnosis of primary melanoma was 53 years (range 18–76). Females were significantly younger than males at diagnosis of both primary melanoma and lung metastases (mean age at primary 44 ± 14 yrs *vs* 58 ± 14, *p* = 0.02; mean age at LM 50 ± 15 vs 63 ± 14, *p* = 0.04) (Table 1). On CT scan, a single pulmonary nodule was observed in 18 cases (72%) while multiple nodules (*n* = 2–4) were observed in the remaining 7 cases (28%). All patients were surgically treated with curative intent and all of them were free of extra-pulmonary disease and regional lymph node involvement. Lung wedge resection was the commonest surgical procedure (14 cases) whereas lobectomy was performed in 11 patients. Median Breslow thickness (available for 21 patients) was 2.3 mm (range 0.75–5 mm) and the trunk was the most frequent primary location (trunk 72%, arm/leg 20%, other sites 8%) (*p* = 0.01). The median metastasis size was 1.6 cm (range 0.5–7 cm) (Table 1).

**Table 1:** Patient demographics and clinical characteristics of primary melanoma and of MLMs

Parameter	All (*n* = 25)	BRAF (*n* = 13)	NRAS (*n* = 5)	WT/WT (*n* = 7)	2-Group *p* value	3-Group *p* value
**Gender**						
Female	10 (40%)	5 (42%)	2 (43%)	3 (36%)		
Male	15 (60%)	8 (58%)	3 (57%)	4 (64%)		
**Age at primary tumor (yrs)**						
Mean ± ds	52 ± 16	50 ± 16	55 ± 22	55 ± 12		
Median	53	47	62	58		
Range	18–76	25–71	18–76	35–70		
≥60 yrs	9	4	3	2		
<60 yrs	16	9	2	5		
Female (mean ± ds)	44 ± 14	41 ± 9	40 ± 31	51 ± 14	0.02^i^	
Male (mean ± ds)	58 ± 14	56 ± 17	65 ± 12	58 ± 12		
**Age at MLM (yrs)**						
Mean ± ds	58 ± 16	56 ± 16	59 ± 20	59 ± 11		
median	61	54	67	61		
range	23–80	32–76	23–80	42–73		
≥60 yrs	13	6	3	4		
<60 yrs	12	7	2	3		
Female (mean ± ds)	50 ± 15	48 ± 11	45 ± 31	57 ± 11	0.04^i^	
Male (mean ± ds)	63 ± 14	62 ± 17	68 ± 13	61 ± 12		
**Number of LMM**						
Single lesions	18 (72%)	9 (69%)	5 (100%)	4 (57%)		
Multiple lesions (*n* = 2–4)	7 (28%)	4 (31%)	-	3 (43%)		
**Surgical procedure**						
Lung wedge resection	14 (56%)	7 (54%)	3 (60%)	4 (57%)		
Lobectomy	11 (44%)	6 (46%)	2 (40%)	3 (43%)		
**Primary site**						
Trunk	18 (72%)	8 (62%)	4 (80%)	6(86%)		0.01^ii^
Arm/Leg	5 (20%)	3 (23%)	1 (20%)	-		
Other	2 (8%)	2 (15%)	-	1(14%)		
**Breslow Thickness (mm)**						
Assessed	21	11	5	5		
Not Assessed	4	2	-	2		
Mean ± ds	2.3 ± 0.9	2.3 ± 0.1	2.1 ± 0.5	2.7 ± 0.6	0.1^iii^	0.5
Median	2.3	2.3	2.1	2.4		
Range	0.75–5	0.75–5	1.5–2.8	2.3–3.2		
**Size of MLM (cm)**						
Mean ±ds	2.3 ± 1.8	1.9 ± 1.1	3.3 ± 2.2	2.2 ± 2.2		0.2
Median	1.6	2	4.2	1.2		
Range	0.5–7	0.5–4.5	0.8–5.5	0.8–7		
<1.6 cm (%)	13 (52%)	6(46%)	2 (40%)	5 (71%)		
mean	1.0 ± 03	0.9 ± 0.3	0.9 ± 0.1	1.0 ± 0.2		
1.6cm (%)	12 (48%)	7(54%)	3(60%)	2 (29%)		
mean	3.6 ± 1.8	2.7 ± 0.9	4.9 ± 0.7	3.9 ± 2.8	0.006^iv^	0.03^v^

i Female vs Male (All)

ii Trunk vs Arm/Leg *vs* Other (All);

iii NRAS vs WT

iv BRAF vs NRAS

v BRAF vs NRAS vs WT

### BRAF/NRAS/CKIT mutation frequencies

BRAF was altered in 13 lung metastases (52%) with all exhibiting a BRAF^V600E^ mutation type (Table 2). NRAS mutations were detected in 5 specimens (20%), 4 NRAS^Q61R^ and 1 NRAS^Q61K^, and were mutually exclusive with BRAF aberrations. A total of 7 lesions (28%) were wild type for both oncogenes (WT/WT). C-KIT mutations were not found (Table 2).

**Table 2:** BRAF/NRAS/CKIT mutation frequency

Molecular pattern	N° cases	(%)
**BRAF mutation**	13	52
V600E	13	100
**NRAS mutation**	5	20
Q61R	4	80
Q61K	1	20
**WT/WT**	7	28
**CKIT mutation**	0	0

### EGFR gene and chromosome 7 abnormalities

**Figure 1:**
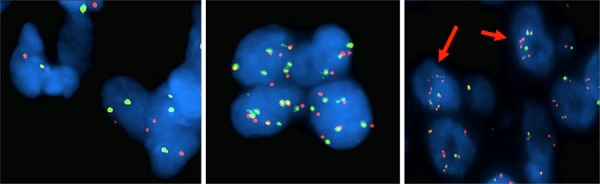
FISH analysis of Chromosome 7 and EGFR copy number alterations EGFR gene-specific probe was labeled with Spectrum Orange (appearing as red signals) and chromosome 7 centromeric probe (Cep7) was labeled with Spectrum Green (appearing as green signals), cell nuclei were stained with blue fluorescent DAPI. Cells with chromosome 7 disomy (left), chromosome 7 polysomy (middle) and EGFR copy number amplification (right, red arrows). Original magnification X100.

FISH analysis was carried out for 17 of 25 specimens. Tumors were classified into 3 groups as described in the methods section. A normal signal was observed in 9 lesions (53%) while aberrant ones were detected in 8 cases (47%) (Table 3, Figure 1). In particular, 3 of 17 cases showed chromosome 7 copy-number-gain (CNG) (17.5%) with a normal EGFR/cep7 ratio, while EGFR specific copy number alterations were recorded in 5 of 17 cases (29, 5%), 3 with EGFR amplification (17.5%) and 2 exhibiting a deletion of the EGFR signal (12%). The three cases with specific EGFR amplification were all BRAF mutated, therefore, concurrent BRAF and EGFR abnormalities were present in 3 of 13 BRAF positive cases (23%). No correlation was observed between NRAS mutation and EGFR copy number alteration (Table 3).

**Table 3:** FISH analysis

		Normal EGFR/Chr 7 ratio		Aberrant EGFR/Chr 7 ratio	
FISH signal	*n* (%)	Disomy *n* (%)	CNG/Polysomy *n* (%)	Amplification *n* (%)	Deletion *n* (%)
**Normal**	9 (53)	9 (100)	-	-	-
BRAF	4 (44)	4 (100)	-	-	-
NRAS	1 (11)	1(100)	-	-	-
WT/WT	4 (44)	4(100)	-	-	-
**Aberrant**	8 (47)	-	3 (37.5)	3 (37.5)	2 (25)
BRAF	5 (60)	-	1(20)	3 (44.5)	1 (11)
NRAS	1(12.5)	-	1(100)	-	-
WT/WT	2 (25)	-	1 (25)	-	1 (25)
**tot**	**17**	**9 (53)**	**3(17.5)**	**3(17.5)**	**2 (12)**

### Patient demographics and pathological characteristics based on BRAF, NRAS and EGFR gene alterations

The cohort was divided into three distinct subgroups based on BRAF and NRAS mutation status: BRAF mutated (from now on referred to as BRAF), NRAS mutated (from now on NRAS) and double wild-type (from now on WT/WT). BRAF patients were slightly younger than NRAS and WT/WT; mean age at primary melanoma and at LM was respectively 50 ± 16 and 56 ± 16 years for BRAF, 55 ± 22 and 59 ± 20 years for NRAS and 55 ± 12 and 59 ± 11 years in WT/WT patients (Table 1). NRAS lesions tended to be larger (mean diameter 3.3 ± 2.2 cm) compared with BRAF (mean diameter 1.9 ± 1.1 cm, *p* = 0.2) but not with respect to WT patients (2.2 ± 2.2 cm; *p* = 0.4). At a diameter cut-off of 1.6 cm, corresponding to the median size of the lesions, the difference among NRAS a BRAF became statistically significant (*p* = 0.006) (Table 1). Gender distribution, age, site and Breslow thickness of primary melanoma was not statistically associated with BRAF or NRAS mutational status. EGFR copy number alterations were not associated with any patient demographics or pathological characteristics.

### Survival analysis

Tumor mutation status was correlated with time to lung metastasis (TLM), overall survival (OS) from primary melanoma as well as relapse-free survival (RFS) and OS after metastasis resection. The median TLM for the 25 pts cohort was 48 months (range 24–192 months) (Table 4). After metastasectomy, 13 of 25 patients (52%) showed recurrence within 60 months from surgery and the median relapse free-survival was 40 months (range 6–156). Median OS after metastasectomy was 52 months (range 12–144) with a 96%, 68% and 28% respective 1, 3 and 5 years survival rate. Median OS from primary melanoma was 108 months (range 60–240) (Table 4).

**Table 4:** Survival analysis

Parameter	All *n* = 25	BRAF *n* = 13	NRAS *n* = 5	WT/WT *n* = 7	NRAS-WT *n* = 20	2-Group *p* value^*^	3-Group *p* value^**^
**TLM (mo)**							
Mean	64	76	48	53	68		0.4
Median	48	48	48	48	48		
Range	24–192	24–192	24–60	24–108	24–192		
Rate LMFI ≤ 5yrs	72%	61%	100%	71%	65%		
Rate LMFI > 5yrs	40%	29%	0%	19%	35%		
**RFS (mo)**							
Mean	49	48	30	64	53	i	0.3
Median	40	36	24	48	42.5		
Range	6–156	6–156	10–52	30–132	6–156		
**OS from LM (mo)**							
Mean	62	63	37	77	68	ii	0.1
Median	52	48	36	70	60		
Range	12–168	12–156	24–52	24–144	12–168		
**OS from LM Rate**							
1 yrs	96%	92%	100%	100%	95%		
3 yrs	68%	69%	40%	86%	75%		
5 yrs	28%	31%	0%	29%	30%		
**OS from primary (mo)**							
Mean	125	138	84	130	136	iii	0.2
Median	108	120	84	96	120		
Range	60–240	96–240	60–96	72–240	72–240		

TLM: time to lung metastasis; RFS: relapse free-survival; OS: overall survival; NRAS-WT: BRAF plus WT/WT; mo=month

* (i) NRAS vs WT/WT *p* = 0.1; NRAS vs NRAS-WT *p* = 0.08 (ii) BRAF vs NRAS *p* = 0.09; NRAS vs WT/WT *p* = 0.057; NRAS vs NRAS-WT *p* = 0.027 (iii) BRAF vs NRAS *p* = 0.0018; NRAS vs NRAS-WT *p* = 0.0008

** BRAF vs NRAS vs WT/WT

**Figure 2:**
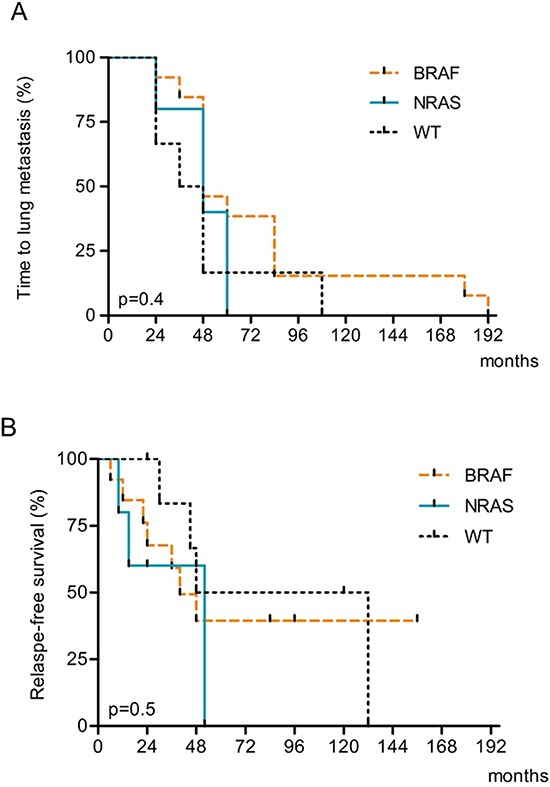
Kaplan-Meier estimates of A Time to lung metastasis (TLM) and **B.** Relapse-free survival (RFS) after lung metastasis resection according to BRAF and NRAS mutation status.

**Figure 3:**
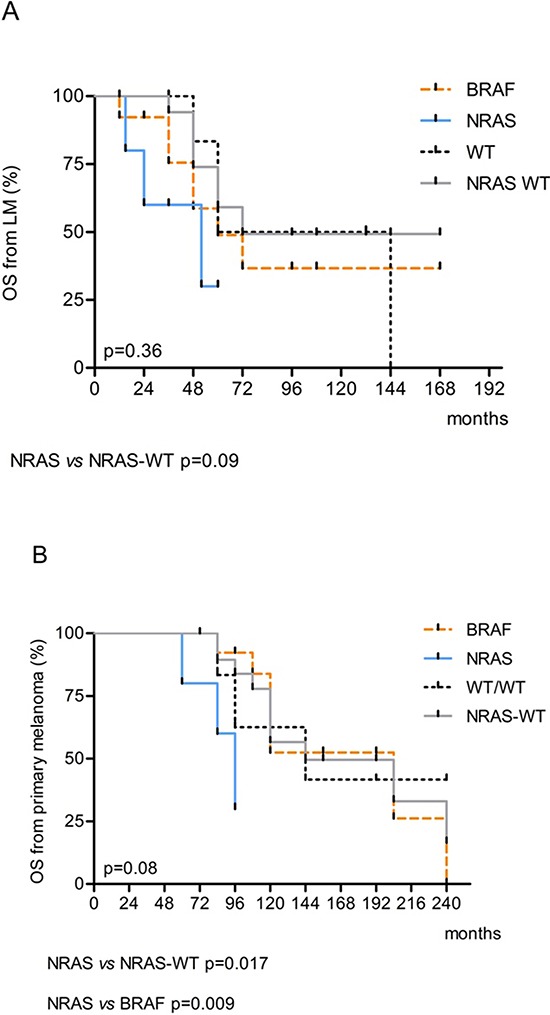
Kaplan-Meier estimates of A Overall Survival (OS) from lung metastasis and **B.** OS from primary tumor according to BRAF and NRAS mutations.

No significant differences in terms of relapse-free-survival after primary melanoma (TLM) or after metastasectomy (RFS) were recorded among the NRAS, BRAF and WT/WT subgroups respect to each other (Table 4, Figure 2A and 2B). Then, we evaluated NRAS-mutated versus NRAS-WT (BRAF-mutated and WT/WT combined) and found a borderline significance in mean RFS after metastasectomy (NRAS-mutated versus NRAS-WT; 30 vs 53 months, *p* = 0.08) (Table 4). Same trend was evident in terms of a shorter OS from metastasectomy in NRAS patients with respect to NRAS-WT (BRAF plus WT/WT combined) (*p* = 0.09) that became significant when OS from the primary tumor was considered (*p* = 0.017) (Table 4; Figure 3A and 3B). Moreover, the multivariate Cox regression analysis, revealed that NRAS mutation was the only predictive factor of adverse survival (*p* = 0.039, OR = 5.5 (1.1–27.6). No difference in survival was observed based on specific EGFR copy number changes.

## DISCUSSION

In this study we performed the molecular appraisal of BRAF, NRAS, CKIT and EGFR genes altogether in a highly selected cohort of melanoma patients with lung metastases, that had been surgically treated with curative intent. No systemic or radiation therapies had been administered to the entire cohort before or after surgery. The frequency of BRAF (52%) and NRAS (20%) mutations was in line with the rates reported for other visceral melanoma metastatic sites [29–34] while no CKIT gene alterations were found. This was in accord with the main derivation of our cohort from primary melanoma of the trunk where only low frequency (2%) of CKIT mutations have been previously documented [22–23]. Also in agreement with previous studies, with respect to age at onset of both, primary and metastasis, females were significantly younger than males regardless of BRAF and NRAS mutation status [30–34]. Breslow thickness of primary melanoma was not significantly associated with BRAF or NRAS gene alterations.

Chromosome 7/EGFR copy number alterations have been studied in primary and metastatic melanoma and speculated to play a role in disease progression [35]. In line with these data our FISH analysis revealed a 47% overall rate of EGFR/chromosome 7 aberrations. Intriguingly, 3 of the 13 BRAF positive cases (23%) displayed a concomitant EGFR copy number amplification. This observation can be relevant in the light of the following data that link EGFR with biologic-therapy resistance: (i) ectopic expression of EGFR in melanoma cells is sufficient to cause resistance to PLX4032 (vemurafenib), a specific small-molecule BRAF inhibitor [36]; (ii) a rapid feedback activation of EGFR can support continued proliferation in the presence of BRAF (V600E) inhibition [36]; (iii) EGFR inhibition blocked proliferation and invasion of BRAFV600E resistant melanoma [37]; (iv) demethylation of EGFR regulatory DNA elements has been observed in cutaneous melanomas resistant to BRAF inhibition [38] and finally (v) a melanoma subtype with intrinsic resistance to BRAF inhibition has been identified that was associated with differential EGFR and ERBB3 expression [39]. With respect to the above observations one could therefore speculate that EGFR amplification might explain, at least in part, the lack of response observed in approximately 20% of patients with melanoma harboring BRAFV600E mutations.

Patients with pulmonary metastases show a better survival rate than individuals affected by metastases to other visceral sites [40] and a growing number of studies have shown that pulmonary metastectomy dramatically improves survival [8, 41–44]. Accordingly, in our study, the survival rate registered for the all cohort of LMM patients was excellent with a median relapse-free and overall survival after surgery of respectively 40 and 52 months that was slightly higher than the ones reported before [8, 45]. Our results probably reflects the accurate selection of patients eligible for surgical excision and/or an intrinsic more favorable outcome linked to a less aggressive disease biology. In this context, our survival analyses intriguingly indicated a trend to a worse prognosis for NRAS mutated patients. In particular, compared with BRAF and WT individuals, all NRAS patients developed lung metastases within 5 years from the primary, tended to carry pulmonary lesions of larger size and showed shorter survival time after lung metastasis resection (Table 4). In addition, the mean OS evaluation from primary melanoma to death or last follow-up, revealed significant differences among NRAS and BRAF patients, as well as among NRAS and their wild-type counterparts and, finally, the multivariate Cox regression analysis showed that NRAS mutation was the only predictive factor of shorter survival from primary melanoma. An adverse prognostic role of NRAS mutations has been already speculated in previous studies. Patients with NRAS mutant melanomas had thicker tumors at presentation and these tumors had greater rates of mitosis than BRAF mutant and WT melanoma [30, 46, 47]. Shorter survival from initial melanoma diagnosis for NRAS patients compared to WT has been reported [30, 46, 47], and NRAS mutation status has been identified as an independent predictor of shorter survival after a diagnosis of stage IV melanoma [46]. From a therapeutic standpoint novel exciting options exist for improving the overall survival of NRAS-driven melanomas. In particular, MEK162, a MEK1/2-inhibitor, was recently proposed as the first type of target therapy to show activity in patients with NRAS-dependent melanoma and, in addition, individuals with similar molecular characteristics were found to be uniquely sensitive to CMET inhibition, thus providing a new rationale for therapeutic targeting of CMET in this patient cohort [48, 49]. Moreover, it was recently reported that a local administration of low-dose IL-2 through inhalation (lh-IL-2) might offer an effective and safe treatment option for lung metastases in melanoma patients and lh-IL-2 may have a prophylactic potential to prevent recurrence to the lung after pulmonary melanoma metastasectomy [50]. Intriguingly, in an independent study, NRAS status was suggested as a new candidate biomarker for selecting melanoma patients for high-dose interleukin-2 treatment (HD IL-2) [51].

This work has one major limitation consisting in the small number of patients enrolled during a 12 years period. This small accrual was mainly due to the extreme rarity of application of the intervention of surgery for the treatment of melanoma lung metastases, and the strict parameters utilized to select the population under molecular study including the lung metastasis as the first manifestation of metastatic disease and the absence of systemic and radiation treatment. Notwithstanding this weakness, we believe that the present investigation, based on a small but carefully selected cohort of pulmonary metastatic melanoma, represents a useful reference and provides several clues concerning the lung as a specific site of melanoma disease that may be highly relevant to identify melanoma patients with diverse prognostic features and therapeutic options.

## MATERIALS AND METHODS

### Tumor samples and patients

The study cohort consisted of 25 pulmonary metastasis specimens obtained by lobectomy (11) or wedge resection (14) from 25 patients affected by melanoma who underwent metastasectomy with curative intent between years 2000 and 2012 at the San Camillo-Forlanini Hospitals. All patients were free of extra-pulmonary disease and lymph node involvement and, in all cases, pulmonary metastasis was the first sign of metastatic disease. Informed consent was acquired from all patients and the study was approved by the Local Institutional Ethics Committee. For all individuals information concerning the site of primary tumor and date of first ever diagnosis was acquired from medical records. Breslow thickness of the primary tumor was available for 21 cases. No patient underwent systemic or radiation therapy before or after surgery.

### DNA extraction and mutation analysis

4 μm thick sections were lightly stained with haematoxylin and manually microdissected by an expert pathologist (LM). Genomic DNA was extracted using the QIAamp DNA FFPE Tissue kit (Qiagen, Germany) following the manufacturer's protocol and subjected to PCR amplification of BRAF (exon 15), NRAS (exon 3) and CKIT (exon 9, 11, 13 and 17). Mutations were tested by Sanger sequencing using a BigDye Terminator Cycle Sequencing Kit (Applied Biosystems, Austin TX, USA) and an ABI PRISM 310 Genetic Analyzer (Applied Biosystems). Sequencing reactions were performed in forward and reverse orientation.

### Fluorescence *in situ* hybridization analysis

Fluorescence *in situ* hybridization was executed by dual colour FISH assay using 7 centromere-(cep7, SpectrumGreen) and locus specific 7p12 (EGFR locus, SpectrumOrange) probes from Vysis (Vysis Inc. IL, USA). FISH was carried out according to the manufacturer's protocol. An average of 50–100 nuclei were screened per each sample. Categories of FISH abnormalities were defined as follows: (i) EGFR gene deletion: EGFR copy number was less than chromosome 7 centromere (cep7) in more than 15% of nuclei; (ii) Chromosome 7 copy number gain (CNG): EGFR/cep7 ratio =1, but the cep7 signals were > 2 per nucleus in more than 15% of nuclei; (iii) EGFR amplification: EGFR/cep7 ratio > 2.2 in more than 15% of screened nuclei.

### Statistical analysis

Statistical analyses were performed using STATISTICA 7 software (Stat Soft, Inc., Tulsa, Okla., USA). Continuous variables were reported as mean, median, and standard deviation while categorical variables as number (n) and percentage (%). For all patients, clinical and pathological features were tested for association with BRAF and NRAS mutation status using *t*-test and non parametric Mann Whitney or Kruskal Wallis tests for continuous variable and Fisher's exact test for categorical variables. Time to lung metastasis (TLM) was defined as the time from primary melanoma to diagnosis of lung metastasis, while relapse-free survival (RFS) was estimated from metastasectomy to first evidence of relapse. Analysis was also made comparing overall survival (OS) from the date of diagnosis of primary melanoma or lung metastasis to death/last follow-up. Survival analyses were performed with the Kaplan-Meier survival curve and compared using the Log-Rank test (Graphpad 5 software). The Cox proportional hazards model was used to determine the significance of variables in predicting adverse factor for survival introducing in the model patient's age, gender, BRAF and NRAS mutations. All statistical tests were performed 2-sided and a *p*-value of < 0.05 was considered statistically significant.
